# Supplementary material for: Neonatal Lead (Pb) Exposure and DNA Methylation Profiles in Dried Bloodspots
Source: Int J Environ Res Public Health. 2020 Sep 17;17(18):6775. doi: 10.3390/ijerph17186775 (PMC7559513; doi:10.3390/ijerph17186775)
Supplement: Supplementary file 1 [file ijerph-17-06775-s001.pdf]

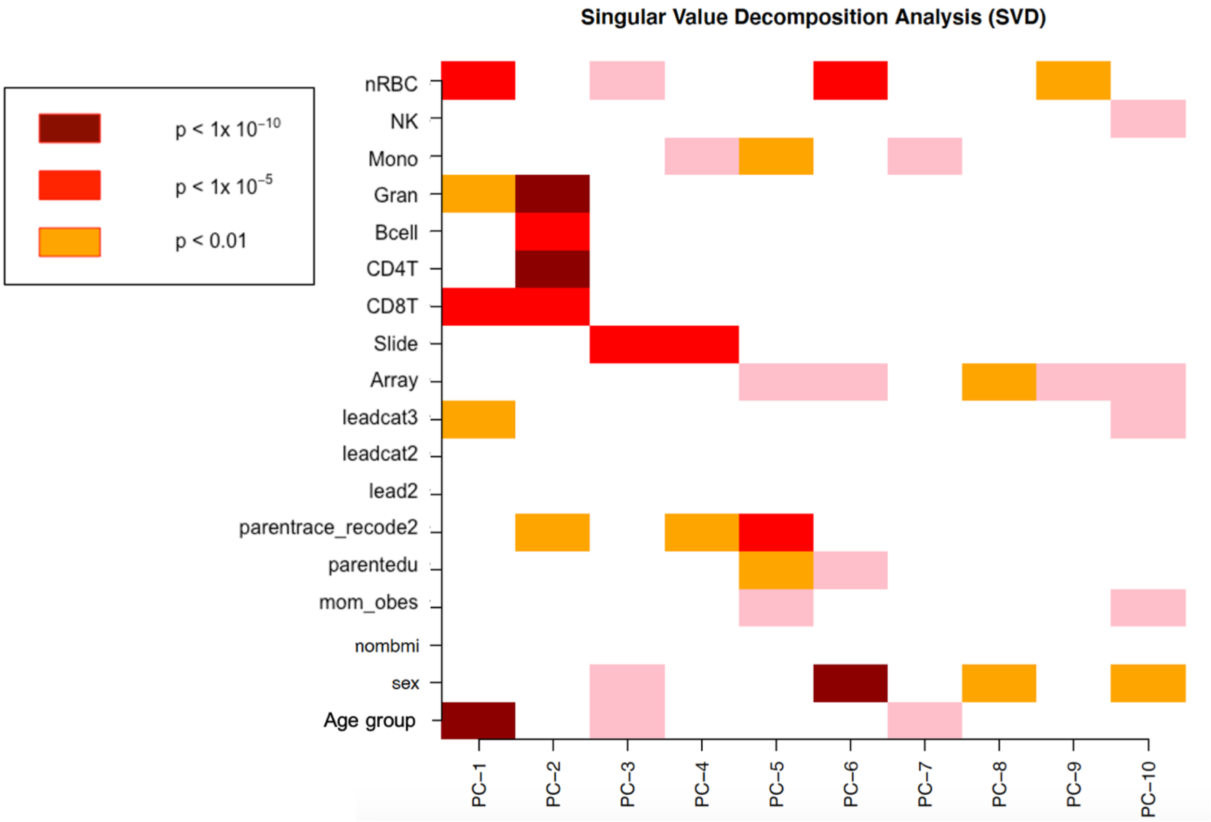

Supplemental Figure 1.

Singular Value Decomposition Analysis (SVD) was conducted to identify covariates associated with DNA methylation data. We used the R package ChAMP to perform SVD to identify technical or biological covariates associated with principle components of the entire DNA methylation dataset. In this analysis, a Kruskal-Wallis test for categorical covariates or a linear model for continuous covariates are used to test associations between covariates and each of the principal components of the DNA methylation beta matrix.

Supplemental Table 1. Estimated cell type percentages and bloodspot Pb by recruitment group. Mean ± SD is listed for recruitment groups 1, 2, and 3. ANOVA was used to test whether cell type proportions were different across groups.

	**Group 1 (n=29)**	**Group 2 (n=25)**	**Group 3 (n=42)**	**ANOVA p-value**
Nucleated Red Blood Cells	3.7 ± 0.02	3.4 ± 0.04	5.1 ± 0.04	**0.0007**
CD8T	6.4 ± 0.04	6.7 ± 0.18	8.7 ± 0.11	**0.005**
CD4T	19.5 ± 0.27	19.8 ± 0.55	19 ± 0.47	0.88

B cells	6.3 ± 0.06	6.2 ± 0.15	5.9 ± 0.05	0.85
Monocytes	9 ± 0.04	9.4 ± 0.1	8.7 ± 0.03	0.51
Granulocytes	58.3 ± 0.44	57.5 ± 1.82	55.8 ± 1.1	0.59
Natural Killer Cells	0 ± 0	0 ± 0	0.02 ± 0	0.53
Bloodspot Pb (μg/dL)	0.65 ± 0.52	0.75 ± 1.18	0.88 ± 0.59	0.51

SD, standard deviation

Cell types estimated in archived neonatal samples, but groups defined by when children were initially enrolled in the study. Group 1, children 12–24 months old; Group 2, children 3–5.99 years old; and Group 3, children 10–12.99 years old

Supplemental Table 2. Differentially Methylated CpG Sites by Pb without cell type adjustment (q<0.2)

Probe ID	Position	Gene Name	Relationship to CpG Island	Estimate (per ug/dL Pb)	SE of Estimate	Average % Methylation at the CpG Site	Raw p-value	q-value
cg03744954	chr7:23637556	*CCDC126*	Island	0.014	0.002	2.2%	2.95E-09	0.0004
cg11961702	chr10:1130138	*WDR37*	Open Sea	-0.008	0.001	98.1%	5.99E-09	0.0004
cg09489281	chr5:43604149	*NNT*	S. Shore	0.035	0.005	5.1%	4.28E-09	0.0004
cg08236836	chr2:95613608		Open Sea	-0.023	0.004	98.4%	6.27E-09	0.0004
cg06157837	chr16:50130934	*HEATR3*	Open Sea	-0.025	0.004	92.7%	6.83E-09	0.0004
cg12819470	chr7:18013541		Open Sea	-0.028	0.004	94.1%	4.28E-09	0.0004
cg00694932	chr22:49029825	*FAM19A5*	Open Sea	-0.034	0.005	87.6%	1.29E-09	0.0004
cg12666827	chr17:81043176	*METRNL*	Island	-0.032	0.005	92.1%	1.24E-09	0.0004
cg09980056	chr6:52366854	*TRAM2*	Open Sea	-0.034	0.005	95.0%	6.55E-09	0.0004
cg11877273	chr1:29182535	*OPRD1*	Open Sea	-0.026	0.004	94.1%	5.33E-09	0.0004
cg16393928	chr1:3135836	*PRDM16*	Open Sea	-0.031	0.005	90.8%	3.24E-09	0.0004
cg27500206	chr11:4600140	*C11orf40*	Open Sea	-0.038	0.006	90.8%	3.47E-09	0.0004
cg25104648	chr18:18566146	*ROCK1*	Open Sea	-0.028	0.004	95.0%	7.58E-09	0.0005
cg25198485	chr4:58086247		Open Sea	-0.026	0.004	92.2%	1.07E-08	0.0006
cg25968149	chr3:174255312		Open Sea	-0.025	0.004	92.9%	1.08E-08	0.0006
cg10174926	chr14:72818968	*RGS6*	Open Sea	-0.024	0.004	93.0%	1.32E-08	0.0007
cg05355328	chr19:33096524	*ANKRD27*	Island	-0.025	0.004	94.2%	1.74E-08	0.0007
cg17533118	chr2:204664739		Open Sea	-0.034	0.006	88.3%	1.75E-08	0.0007
cg06019448	chr4:190417346		Open Sea	-0.030	0.005	90.5%	1.50E-08	0.0007
cg05026437	chr3:99527476	*MIR548G*	Open Sea	-0.027	0.004	92.5%	1.59E-08	0.0007
cg09413029	chr10:97152467	*SORBS1*	Open Sea	-0.027	0.004	93.0%	1.92E-08	0.0007
cg14645844	chr8:123831620	*ZHX2*	Open Sea	-0.030	0.005	93.7%	2.34E-08	0.0008
cg14170794	chr18:76636062		N. Shelf	-0.025	0.004	93.8%	3.07E-08	0.0011
cg01451253	chr17:47014634	*SNF8*	Open Sea	-0.029	0.005	94.2%	3.34E-08	0.0011
cg14810301	chr5:88169099	*MEF2C*	Open Sea	-0.023	0.004	93.3%	4.42E-08	0.0014
cg23649301	chr2:26689647	*OTOF*	Open Sea	-0.030	0.005	92.7%	4.67E-08	0.0014

cg22667391	chr12:57843848	*INHBC*	Open Sea	-0.035	0.006	89.8%	5.10E-08	0.0015
cg14488043	chr11:8788397	*ST5*	Open Sea	-0.029	0.005	91.7%	5.62E-08	0.0016
cg04843855	chr19:17176218	*HAUS8*	Open Sea	-0.026	0.004	90.6%	5.99E-08	0.0016
cg01872216	chr14:54430382		Island	0.006	0.001	2.5%	7.47E-08	0.0020
cg16849089	chr2:23458339		Open Sea	-0.024	0.004	93.9%	8.21E-08	0.0020
cg20383654	chr1:39490535	*NDUFS5*	Open Sea	-0.021	0.004	93.0%	8.30E-08	0.0020
cg04897245	chr4:110616638	*CASP6*	Open Sea	-0.026	0.005	94.4%	8.06E-08	0.0020
cg22581270	chr16:3193156	*CASP16P*	S. Shore	-0.027	0.005	83.7%	1.06E-07	0.0025
cg03270919	chr1:16450404		Open Sea	-0.027	0.005	92.1%	1.11E-07	0.0025
cg23724374	chr6:150973466	*PLEKHG1*	Open Sea	-0.021	0.004	89.7%	1.16E-07	0.0025
cg01532946	chr8:28556590	*NA*	N. Shore	-0.042	0.007	88.5%	1.24E-07	0.0027
cg04828493	chr13:111328783	*CARS2*	N. Shelf	-0.009	0.002	94.8%	1.66E-07	0.0035
cg16926323	chr15:75630342	*COMMD4*	S. Shore	-0.014	0.003	92.5%	1.83E-07	0.0036
cg12101340	chr13:53972317		Open Sea	-0.027	0.005	90.5%	1.83E-07	0.0036
cg20120848	chr6:125918933		Open Sea	-0.011	0.002	91.4%	1.97E-07	0.0038
cg07705707	chr17:77259848	*RBFOX3*	Open Sea	-0.031	0.006	89.8%	2.47E-07	0.0046
cg23828375	chr9:136694260	*VAV2*	Open Sea	-0.009	0.002	92.4%	3.23E-07	0.0059
cg23721440	chr4:39633825	*SMIM14*	Open Sea	-0.010	0.002	92.2%	3.75E-07	0.0067
cg07132558	chr2:2842206		Open Sea	-0.027	0.005	85.9%	4.42E-07	0.0077
cg19631815	chr12:113528791	*DTX1*	N. Shelf	-0.003	0.001	97.7%	4.93E-07	0.0083
cg04368332	chr12:6756663	*ACRBP*	Island	0.008	0.002	3.8%	4.90E-07	0.0083
cg17574471	chr4:1834678	*LETM1*	Open Sea	-0.003	0.001	97.7%	5.36E-07	0.0088
cg14561596	chr4:133045178		Open Sea	-0.012	0.002	94.9%	5.90E-07	0.0095
cg07366047	chr2:135921205	*RAB3GAP1*	Open Sea	-0.031	0.006	94.1%	6.31E-07	0.0100
cg06051201	chr1:53925306	*DMRTB1*	Island	-0.025	0.005	95.1%	6.85E-07	0.0106
cg17274640	chr11:111637274	*PPP2R1B*	S. Shore	0.007	0.001	2.8%	7.92E-07	0.0118
cg08771084	chr10:112193855		Open Sea	-0.017	0.003	84.8%	7.84E-07	0.0118
cg13798146	chr15:83875703	*HDGFRP3*	Island	0.003	0.001	1.1%	8.96E-07	0.0131
cg20396385	chr7:1855709	*MAD1L1*	Open Sea	-0.009	0.002	94.6%	9.79E-07	0.0141
cg07431286	chr5:72526496		Island	0.003	0.001	3.1%	1.05E-06	0.0148
cg06160789	chr18:43430151	*EPG5*	Open Sea	-0.030	0.006	89.4%	1.16E-06	0.0160
cg01162224	chr16:87855236		Open Sea	0.019	0.004	5.2%	1.26E-06	0.0171
cg26291737	chr19:11368633	*DOCK6*	Open Sea	-0.023	0.005	72.5%	1.33E-06	0.0178
cg27169709	chr2:109248169	*LIMS1*	Open Sea	-0.008	0.002	92.3%	1.42E-06	0.0185
cg12089032	chr8:72881203		Open Sea	-0.037	0.007	91.6%	1.43E-06	0.0185
cg20633276	chr1:53925299	*DMRTB1*	Island	-0.025	0.005	94.9%	1.68E-06	0.0214
cg10896152	chr6:47043561		Open Sea	-0.035	0.007	88.8%	1.84E-06	0.0230
cg24320398	chr13:47472158	*HTR2A*	Open Sea	-0.014	0.003	96.3%	1.93E-06	0.0238
cg09049063	chr5:179298526	*TBC1D9B*	Open Sea	-0.033	0.007	92.3%	1.99E-06	0.0242
cg00148035	chr12:111543137	*CUX2*	Open Sea	-0.023	0.004	82.7%	2.06E-06	0.0247

cg17203448	chr21:30115266		Open Sea	-0.023	0.005	90.1%	2.58E-06	0.0304
cg26691849	chr7:50340218		N. Shelf	-0.020	0.004	76.9%	3.50E-06	0.0406
cg06443678	chr17:55785087		Open Sea	-0.008	0.002	95.8%	3.85E-06	0.0437
cg11828218	chr8:120593576	*ENPP2*	Open Sea	-0.015	0.003	88.1%	3.87E-06	0.0437
cg15406566	chr16:4606279	*C16orf96*	Open Sea	0.010	0.002	87.4%	3.97E-06	0.0441
cg04831943	chr6:159519421		Open Sea	-0.010	0.002	92.2%	4.42E-06	0.0485
cg11865553	chr15:77376250		Open Sea	0.005	0.001	2.1%	4.81E-06	0.0520
cg00106808	chr2:3045057		Open Sea	-0.025	0.005	85.6%	5.79E-06	0.0617
cg19041743	chr5:160170132	*ATP10B*	Open Sea	-0.019	0.004	90.9%	6.45E-06	0.0679
cg09809932	chr1:6515597	*ESPN*	Island	0.011	0.002	1.9%	7.04E-06	0.0728
cg08157118	chr8:75547027	*MIR2052HG*	Open Sea	-0.028	0.006	89.7%	7.11E-06	0.0728
cg08048635	chr4:170204675		Open Sea	-0.031	0.007	94.0%	7.26E-06	0.0735
cg13644497	chr5:155371995		Open Sea	-0.019	0.004	88.8%	7.43E-06	0.0742
cg00275425	chr3:195342138		N. Shelf	-0.008	0.002	94.9%	8.87E-06	0.0875
cg06016162	chr1:230400054	*GALNT2*	Open Sea	-0.010	0.002	92.5%	9.55E-06	0.0930
cg19244091	chr5:113437065		Open Sea	0.010	0.002	7.4%	9.81E-06	0.0932
cg01101326	chr4:186893075		Open Sea	-0.033	0.007	92.9%	9.72E-06	0.0932
cg16848956	chr11:72464583	*ARAP1*	N. Shore	-0.015	0.003	90.4%	1.03E-05	0.0967
cg16411445	chr21:40145923	*NCRNA00114*	Open Sea	-0.011	0.002	93.7%	1.07E-05	0.0989
cg09358482	chr14:55374534		Open Sea	-0.029	0.006	91.1%	1.08E-05	0.0992
cg00365268	chr5:17240103	*BASP1*	Open Sea	-0.030	0.007	81.7%	1.13E-05	0.1026
cg13673137	chr19:1752327	*ONECUT3*	N. Shore	0.003	0.001	2.3%	1.20E-05	0.1079
cg09454524	chr2:45918306	*PRKCE*	Open Sea	-0.011	0.002	88.1%	1.24E-05	0.1098
cg10042885	chr17:29876518		Island	0.004	0.001	3.1%	1.25E-05	0.1098
cg23217557	chr14:21578158		Open Sea	-0.019	0.004	80.5%	1.31E-05	0.1120
cg04429341	chr2:3261194	*TSSC1*	Open Sea	-0.012	0.003	91.9%	1.35E-05	0.1120
cg01274770	chr17:15804426		Open Sea	-0.009	0.002	91.5%	1.36E-05	0.1120
cg21718868	chr11:65018011		Open Sea	0.009	0.002	4.3%	1.36E-05	0.1120
cg27068385	chr12:122470620	*BCL7A*	Open Sea	-0.011	0.002	90.9%	1.35E-05	0.1120
cg27600432	chr4:13529102		N. Shore	0.003	0.001	2.7%	1.31E-05	0.1120
cg07556911	chr7:2044787	*MAD1L1*	Island	-0.019	0.004	82.2%	1.42E-05	0.1155
cg03196485	chr2:87021117	*CD8A*	S. Shelf	0.037	0.008	59.4%	1.43E-05	0.1155
cg12506121	chr12:50238076	*BCDIN3D*	S. Shore	-0.008	0.002	93.7%	1.55E-05	0.1223
cg10553102	chr5:148626066	*ABLIM3*	Open Sea	-0.034	0.008	89.0%	1.57E-05	0.1223
cg01876183	chr20:43211328	*PKIG*	Open Sea	-0.010	0.002	91.3%	1.56E-05	0.1223
cg27094173	chr11:73371753	*PLEKHB1*	N. Shore	-0.007	0.002	92.9%	1.70E-05	0.1315
cg08568706	chr20:29591086		Open Sea	0.015	0.003	28.4%	1.74E-05	0.1332
cg07844255	chr21:32556964	*TIAM1*	Open Sea	0.009	0.002	6.1%	1.79E-05	0.1343
cg24889694	chr8:101522535		Open Sea	-0.011	0.002	92.6%	1.79E-05	0.1343
cg07777742	chr17:17920655		Open Sea	-0.011	0.002	90.5%	1.85E-05	0.1376

cg14465044	chr19:55699363	*PTPRH*	Open Sea	0.007	0.002	91.5%	1.93E-05	0.1421
cg25704068	chr2:26738759	*OTOF*	Open Sea	-0.031	0.007	91.4%	2.02E-05	0.1479
cg01881476	chr10:70220573	*DNA2*	Open Sea	-0.014	0.003	90.1%	2.06E-05	0.1491
cg01103255	chr2:201753570	*NIF3L1*	Open Sea	0.034	0.008	12.7%	2.10E-05	0.1507
cg18452653	chr8:67527699		S. Shore	-0.008	0.002	92.1%	2.13E-05	0.1512
cg03751055	chr10:131380455	*MGMT*	Open Sea	-0.069	0.016	87.4%	2.16E-05	0.1519
cg02857398	chr13:111305767	*CARS2*	Open Sea	-0.006	0.001	92.9%	2.24E-05	0.1550
cg19741112	chr6:13299692		Open Sea	-0.012	0.003	92.7%	2.22E-05	0.1550
cg15621427	chr1:204283717	*PLEKHA6*	Open Sea	-0.020	0.005	90.5%	2.44E-05	0.1663
cg12822816	chr10:134361019	*INPP5A*	Island	-0.006	0.001	95.3%	2.43E-05	0.1663
cg12359609	chr1:113015058	*WNT2B*	Open Sea	-0.006	0.001	91.5%	2.67E-05	0.1804
cg14317609	chr9:36986006	*PAX5*	Island	0.007	0.002	5.3%	2.80E-05	0.1845
cg05484949	chr17:38708465		Open Sea	-0.011	0.002	94.6%	2.81E-05	0.1845
cg13804141	chr20:56043685		Open Sea	-0.008	0.002	92.9%	2.79E-05	0.1845
cg26902026	chr11:130785536	*SNX19*	N. Shore	0.013	0.003	81.3%	2.96E-05	0.1930
cg25131632	chr10:94549040		Open Sea	-0.011	0.002	94.9%	3.05E-05	0.1975

Supplemental Table 3. CpG sites with More Variability as Pb Exposure Increases, Identified with DiffVar (q<0.2)

Probe ID	Position	Gene Name	Model Estimate	SE of estimate	p-value	q-value
cg27623075	chr11:110318240	*FDX1*	0.073	0.014	5.50E-08	0.04
cg03744954	chr7:23637556	*CCDC126*	0.148	0.026	1.27E-07	0.05
cg04897245	chr4:110616638	*CASP6*	0.160	0.029	2.91E-07	0.08
cg17533118	chr2:204664739		0.162	0.030	5.13E-07	0.10
cg13781041	chr7:143599522	*TCAF1*	0.099	0.019	6.84E-07	0.10
cg13569765	chr11:65141519		0.197	0.040	7.39E-07	0.10
cg04813347	chr2:218937178	*RUFY4*	0.094	0.019	8.92E-07	0.10
cg07366047	chr2:135921205	*RAB3GAP1*	0.216	0.041	1.05E-06	0.10
cg19074254	chr3:11645861	*VGLL4*	0.165	0.033	1.15E-06	0.10
cg20463033	chr12:46663274	*SLC38A1*	0.058	0.011	1.32E-06	0.10
cg00732589	chr11:110299881	*FDX1*	0.082	0.016	1.89E-06	0.13
cg06160789	chr18:43430151	*EPG5*	0.134	0.029	2.04E-06	0.13
cg06096994	chr13:41635664	*WBP4*	0.058	0.011	2.83E-06	0.17
cg00703822	chr4:10145483		0.134	0.027	3.48E-06	0.18
cg10365769	chr4:7589937	*SORCS2*	0.089	0.019	3.50E-06	0.18

A measure of variability is calculated for each CpG in each sample by subtracting out the group mean and taking the absolute deviation. A linear model is then fitted to the absolute deviations.

This tests the null hypothesis that group variances are equal. Deviations (model estimates)

Here models adjusted for cell type proportions, race, and sex.

These estimates are all positive, signifying that with higher Pb exposure, there is more variability
